# Reviewers Index

**DOI:** 10.3400/avd.ri.20-01000

**Published:** 2020-12-25

(The reviewers who completed the first peer-review for manuscripts submitted during Nov 1, 2019–Oct 31, 2020.)

The ten reviewers written in boldface reviewed more than 6 papers.

Annals of Vascular Diseases do thank all the reviewers for their strong support and cooperation.

## A

Adachi, Koichi

Akahane, Masaaki

Akazawa, Hiroshi

Akutsu, Koichi

**Al-Salman, Mussaad M.S.**

Altarazi, Louay

Anai, Hiroshi

Asakura, Toshihisa

Asano, Soichi

Azuma, Nobuyoshi

## B

**Banno, Hiroshi**

**Bashar, Abul Hasan Muhammad**

Bessho, Ryuzo

## C

Cheng, Stephen WK

Chikazawa, Genta

## F

Fletcher, John

Fujii, Takeshiro

Fukuda, Hirotsugu

Fukuda, Ikuo

Fukui, Kozo

Furuyama, Tadashi

## H

Hamamoto, Masaki

Handa, Nobuhiro

Harada, Hirohisa

Haruta, Naoki

Hattori, Tsutomu

Hayashida, Naoki

Hiraoka, Arudo

Hiromatsu, Shinichi

Hosaka, Akihiro

Hoshina, Katsuyuki

Hosoi, Yutaka

Huh, Seung

## I

Ichihashi, Shigeo

Inaba, Masashi

Inoue, Masanori

Inuzuka, Kazunori

Ishibashi, Hiroyuki

Ishikawa, Tetsuya

Ito, Hiroyuki

Ito, Tsutomu

**Iwata, Hirohide**

Iyori, Keiji

Izumi, Yuichi

## J

Jibiki, Masatoshi

Jin, Hyun Joh

## K

Kadohama, Takayuki

Kamal, Dhafer M

Kamiya, Hiroyuki

**Kanaoka, Yuji**

Katsumata, Takahiro

Kawaharada, Nobuyoshi

Kichikawa, Kimihiko

Kitagawa, Takeshi

Kitano, Ikurou

Kobayashi, Masayoshi

Kodama, Akio

**Koizumi, Jun**

Kokubo, Taku

Komai, Hiroyoshi

Kondo, Norihiro

Kritayakirana, Kritaya

Kudo, Toshifumi

Kuma, Sosei

Kunihara, Takashi

Kurabayashi, Masahiko

Kurihara, Nobuhisa

Kurimoto, Yoshihiko

## L

Laohapensang, Kamphol

Lee, Byung-Boong

## M

Maeda, Hideaki

Maeda, Koji

**Masaki, Hisao**

Matsubara, Junichi

Matsubara, Kentaro

Matsubara, Shinobu

Matsuda, Hitoshi

Matsumoto, Takuya

Matsushita, Masahiro

Midorikawa, Hirofumi

Minatoya, Kenji

Mine, Takahiko

Mitsuoka, Hiroshi

Miyata, Tetsuro

Mo, Makoto

**Morikage, Noriyasu**

Morota, Tetsuro

Motomura, Noboru

Murakami, Atsubumi

## N

Nakamura, Takashi

Nakazawa, Tatsu

**Nishibe, Toshiya**

Nishigami, Kazuhiro

Nishimura, Motonobu

Nishimura, Yoshiharu

Nunokawa, Masao

## O

**Obara, Hideaki**

Ogawa, Tomohiro

Ogawa, Yukihisa

Ohhashi, Toshio

Ojiro, Masataka

Okazaki, Jin

Orihashi, Kazumasa

Otani, Norifumi

## P

Pinjala, Ramakrishna

## S

Saiga, Atsushi

Sakuda, Hitoshi

Sano, Masaki

Sato, Osamu

Sato, Tadaya

Satokawa, Hirono

Sawano, Makoto

Seto, Tatsuichiro

Shigematsu, Kunihiro

Shiiya, Norihiko

Shimizu, Tsuyoshi

Shimpo, Hideto

Shindo, Shunya

Sugano, Norihide

Suzuki, Hiroshi

Suzuki, Shinichi

## T

Taguchi, Shinichi

Tajima, Hiroyuki

Takagi, Gen

Takagi, Yasushi

Takahashi, Toshiki

Takano, Hiroshi

Takayama, Katsutoshi

Takayama, Toshio

Takayama, Yutaka

Tanaka, Hiroyuki

Tanemoto, Kazuo

Tateno, Kaoru

Tomaru, Takanobu

Toya, Naoki

Tsukube, Takuro

## U

Ueda, Tatsuo

Ueyama, Keishi

## W

Wada, Hideichi

Wang, Jinsong

Washiyama, Naoki

## Y

Yamamoto, Hiroshi

Yamamoto, Kota

Yasugi, Takumi

Yiu, Wai-ki

Yoshida, Hiroki

Yoshimura, Koichi

Yoshitake, Akihiro

Yoshizumi, Masao

Yunoki, Yasuhiro

Yuri, Koichi

## Z

Zempo, Nobuya

